# Adherence to Guideline-Directed Medical Therapy Target in patients with heart failure and reduced ejection fraction: a cross-sectional study

**DOI:** 10.1590/1516-3180.2023.0315.R2.13082024

**Published:** 2025-05-02

**Authors:** Fábio Figueirêdo Costa, Andréa Karoline Reis Chagas, Anna Cláudia Monteiro Luz Santos, Lívia Brito Oliveira, Alex Cleber Improta-Caria, Adriana Lopes Latado, Roque Aras

**Affiliations:** I Department of Cardiology, Hospital Universitário Professor Edgard Santos (HUPES), Universidade Federal da Bahia (UFBA), Empresa Brasileira de Serviços Hospitalares (EBSERH), Salvador, BA, Brazil.; II Department of Cardiology, Hospital Universitário Professor Edgard Santos (HUPES), Universidade Federal da Bahia (UFBA), Empresa Brasileira de Serviços Hospitalares (EBSERH), Salvador, BA, Brazil.; III Department of Cardiology, Hospital Universitário Professor Edgard Santos (HUPES), Universidade Federal da Bahia (UFBA), Empresa Brasileira de Serviços Hospitalares (EBSERH), Salvador, BA, Brazil.; IV Research and Technological Innovation Management Sector, Hospital Universitário Professor Edgard Santos (HUPES), Salvador, BA, Brazil.; V Postdoctoral Researcher, School of Physical Education and Sport, Laboratory of Biochemistry and Molecular Biology of the Exercise, Universidade de São Paulo (USP), São Paulo, SP, Brazil.; VI Department of Internal Medicine, Faculty of Medicine, Hospital Universitário Professor Edgard Santos (HUPES), Universidade Federal da Bahia (UFBA), Salvador, BA, Brazil.; VII Department of Internal Medicine, Faculty of Medicine, Hospital Universitário Professor Edgard Santos (HUPES), Universidade Federal da Bahia (UFBA), Salvador, BA, Brazil.

**Keywords:** Heart failure., Drug prescriptions., Medication adherence, Cardiac failure, Medical prescription, Drug therapies, Guideline-directed medical therapy

## Abstract

**BACKGROUND:**

Heart failure with reduced ejection fraction (HFrEF) represents a compelling cause of hospital morbidity and mortality in Brazil. There is low adherence to guideline-directed medical therapy (GDMT), which in turn, can result in higher morbidity and mortality.

**OBJECTIVES:**

The present study aims to evaluate adherence to GDMT in patients with HFrEF in a Brazilian University hospital service.

**DESIGN AND SETTINGS:**

Observational, cross-sectional, single-center study conducted at the Hospital Universitário Professor Edgard Santos (HUPES), Salvador, BA, Brazil.

**METHODS:**

The study was conducted with convenience sampling at the cardiology outpatient clinic of a university hospital service. Patients with left ventricular ejection fraction (LVEF) < 40% who had reverse remodeling were excluded.

**RESULTS:**

289 patients were included, with mean age 63 years, 54.7% were male, 56,4% mixed-race and 27,7% had Chagasic cardiomyopathy. 93.1% were prescribed ACEi, ARB or ARNi, 95.8% betablockers, 69.2% spironolactone and 8% the combination hydralazine/isosorbide-dinitrate. 71,7% were using enalapril, losartan or ARNi above 50% of GDMT target doses; 81,2% were using beta-blockers and 100% were using spironolactone. Only 21,2% were prescribed GDMT target doses of enalapril, losartan or ARNi and 52,3% of beta-blockers. 98,5% of spironolactone prescriptions reached GDMT target doses.

**CONCLUSIONS:**

We found high frequencies of prescription of GDMT for HFrEF, considering the therapeutic goals recommended by cardiology guidelines, but, prescription of target doses were low in ACEi, ARB or ARNi and beta-blockers.

## INTRODUCTION

Cardiovascular diseases are the main causes of morbidity and mortality in Brazil and worldwide, and heart failure (HF) is one of its main representatives.^1,2^ With population aging in our country, the costs, the need for hospital beds and trained health care team to properly treat this condition will be increasing, because the prevalence of the disease increases with aging.^1-4^ In 2023, there were 206,230 hospitalizations for HF in Brazil with 24,203 deaths, according to DATASUS.^1^ These hospitalizations represented a cost of R$ 481.152.294,08.^1^ In patients with severe HF, only 35% may be alive after 5 years^4^ and in the Brazilian BREATHE^2^ registry, the in-hospital mortality rate was as high as 12.6%.

The ideal pharmacological therapy for HF follows the clinical characterization of the syndrome and/or the results of complementary diagnostic methods.^4^ Patients with HF and reduced left ventricular ejection fraction (HFrEF) - left ventricular ejection fraction (LVEF) < 40% - and who are symptomatic, are stratified in functional classes by the New York Heart Association (NYHA). There are drug therapies effective in reducing the incidence of fatal and non-fatal outcomes^4-7^ in patients in NYHA I to IV. Thus, the use of guideline-directed medical therapy (GDMT) is strongly recommended.^4-7^

Angiotensin-converting enzyme inhibitors (ACEi), angiotensin (II) receptor blockers (ARB) in those intolerants to ACEi, beta-blockers (namelly carvedilol, bisoprolol and metoprolol succinate, or nebivolol in the elderly);^8^ and mineralocorticoid receptor antagonists (MRA) constitute the initial drug triad recommended for patients with HFrEF.^4^ The hydralazine-dinitrate combination, in the Brazilian Heart Failure (HF) guideline, also receives a grade I recommendation for patients with HFrEF who can´t tolerate ACEi and/or ARB and for self-declared Afro-Americans who remain symptomatic despite optimal clinical therapy.^4^ Most recently, angiotensin receptor-neprilysin inhibitors (ARNi) and sodium-glucose cotransporter-2 inhibitors (SGLT2i) have also been incorporated to the therapeutic arsenal of drugs capable to reduce mortality and morbidity in HFrEF.^5-7^

Although the landmark studies of ACEi, beta-blockers and MRA have already been published for more than two decades^9-16^ there is low adherence to GDMT worldwilde.^2,17-20^ This low adherence is associated with higher all-cause mortality and higher rates of hospital admissions.^18^ Additionally, it is worth to know that the prescription of at least 50% of target doses of GDMT is associated with reduced clinical outcomes.^18^

## OBJECTIVE

This study aims to evaluate the adherence of medical prescriptions to GDMT in patients with HFrEF treated in an outpatient center of reference for medium and high complexity in cardiovascular care, considering also, their recommended target doses.^4-7^ Secondarily, we evaluated the prescription of other pharmacological therapies for HFrEF that have lower degrees of recommendation^4-7^ and prescriptions according to LVEF, sex and age.

## METHODS

### Study design and site

It is an observational, cross-sectional, predominantly descriptive, single-center study. The research site was the outpatient section of the Cardiology service of a University Hospital Complex, located in northeastern Brazil. The health institution cares for patients from the National Health System (in Portuguese Sistema Único de Saúde – SUS) and comprises cardiology care in general cardiology, valvopathies, myocardiopathies, arrhythmias, coronary artery disease, cardiac devices, perioperative, interconsultations, cardio-oncology, cardiac surgery and clinical research.

### Sampling and Study Population

Sampling was by convenience and composed of patients who was seen through electronic medical records in the cardiology outpatient clinics.

We included patients older than 18 years with LVEF < 40% (by echocardiogram, nuclear medicine, magnetic resonance or contrast ventriculography), NYHA class II to IV and who had properly and available electronic clinical and prescription records.

Exclusion criteria: patients with prior LVEF < 40% who had heart failure with improved ejection fraction (HFimpEF).

### Procedures of the study

On a weekly basis, from November 2018 to August 2019, the researchers (cardiology physicians at the institution) obtained the lists of cardiology care performed and, in possession of these lists, accessed the electronic medical records of the patients seen, filling out the data collection form specifically designed and approved for the study. The data collection included demographic variables, heart failure characteristics, laboratory and imaging tests, in addition to variables related to the prescriptions pattern.

### Outcomes of interest

#### Primary Outcome

As the primary outcome of the study, we defined the percentage of patients with HFrEF treated in the outpatient clinics of the cardiology service who received GDMT with class I recommendation and whether the doses prescribed reached the target dose or at least 50% of the recommended target doses. For this analysis, the recommendations on drug doses contained in the 2018 Brazilian Chronic and Acute Heart Failure Guideline of the Brazilian Society of Cardiology (SBC)^4^ were applied. For carvedilol, the reference target dose used was the one from US Carvedilol studies^12,13^ i.e., 50 mg/day for most patients (weight < 85Kg).

#### Secondary Outcomes

The secondary outcomes evaluated were the percentage of use of medications that do not necessarily alter mortality, but that can positively alter morbidity and the association, of exploratory nature, between the use of specific classes of drugs with grade I of recommendation, according to the clinical guidelines,^4-7^ and the variables age, sex, race/ethnicity, etiology and NYHA functional classification, Diabetes Mellitus (DM), chronic kidney disease (CKD) and atrial fibrillation (AF).

We also did an analysis of optimal and suboptimal adherence to GDMT, where three levels of adherence are defined: good adherence (use of three indicated medications from the triad – score 1), moderate adherence (use of more than half of the indicated medications – score > 0.5) and poor adherence (use of less than half of the indicated medications – score < 0.5)^20^ and a subanalysis by sex, age (≥ 75 e < 75) and LVEF (< 30% e ≥ 30%).

### Statistical Analysis

Statistical analysis was performed with R for Windows software, version 4.1, which is freely accessible.^21^ For descriptive statistics, categorical variables were described as proportions and quantitative variables were described as means (standard deviation) and medians (interquartile range), according to the normality of the data. The normality of quantitative variables was assessed by the Shapiro-Wilk statistical test and by the distribution characteristics (kurtosis and skewness). For exploratory analytical statistics, Student’s t-test was used for comparison of means, or non-parametric test for medians. Chi-square and Fisher’s exact tests were used for the bivariate analyses between categorical variables. Multivariate models by unconditional logistic regression were used to assess the potentially independent association between prognostic variables and prescription pattern for HFrEF, with focus on the target doses of the drugs. The measure of association obtained was the odds ratio (OR), with their respective 95% confidence intervals. For the purpose of statistical inference, a p value of less than 5%, two-tailed, was considered statistically significant.

### Ethical aspects

The present study complies with the ethical principles involving research in humans, as guided by resolution no. 466/2012, of the National Health Council, and was approved by the Research Ethics Committee of the institution in CAAE: 01203618.4.0000.0049 on October 29, 2018.

## RESULTS

Between November 2018 and August 2019, 289 patients were included in the study. The mean age was 63 years, 54.7% were male, 35.3% were diabetic, and 28% had CKD (Table 1). The majority self-declared mixed race/ethnicity (56.4%), followed by Afro-Americans (30.1%) and Caucasians (11.1%). When analyzing the distribution of age, race/ethnicity and comorbidities by ejection fraction (LVEF < 30% and LVEF > 30%), we found no significant differences. Male sex where more prevalent in those with severe HF (Table 1).

**Table 1:** Baseline characteristics of the sample

	Total (n = 289)	LVEF < 30% (n = 105)	LVEF = 30% (n = 184)
Age in years - mean (SD)	63 (12.4)	61 (13.6)	64 (11.7)
Male Sex % (n)	54.7% (158)	63.8% (67)	49.5% (91)
Race/Ethnicity % (n)			
Mixed Race	56.4% (163)	61.9% (65)	53.3% (98)
Afro American	30.1% (87)	24.8% (26)	33.1% (61)
Caucasian	11.1% (32)	10.4% (11)	11.4% (21)
Unavailable	2.4% (7)	2.9% (3)	2.2% (4)
HF etiology % (n)			
Chagas Disease	27.7% (80)	27.6% (29)	27.7% (51)
Ischemic	26.6% (77)	25.7% (27)	27.2% (50)
Hypertensive	17% (49)	15.2% (16)	17.9% (33)
Idiopathic	9.3% (27)	11.4% (12)	8.2% (15)
Valvar	5.5% (16)	1% (1)	8.2% (15)
Inflammatory	5.2% (15)	9.5% (10)	2.7% (5)
Alcoholic	4.8% (14)	7.6% (8)	3.4% (6)
Other**	3.8% (11)	1.9% (2)	4.4% (8)
Mean LVEF % (SD)	31.5 (7)	23.4 (4.2)	36.1 (3.1)
NYHA status % (n)			
I	1% (3)	0% (0)	1.6 (3)
II	63.7% (184)	48.6% (51)	72.3% (133)
III	30.1% (87)	40% (42)	24.5% (45)
IV	5.2% (15)	11.4% (12)	1.6% (3)
Hypertension % (n)	66.8% (193)	64.8% (68)	67.9% (125)
Diabetes Mellitus % (n)	35.3% (102)	27.6% (29)	39.7% (73)
Dyslipidemia % (n)	59.9% (173)	48.6% (51)	66.5% (122)
AF/Flutter % (n)	23.2% (67)	22.9% (24)	23.4% (43)
Chronic kidney disease % (n)	28% (81)	33.3% (35)	25% (46)
BMI Kg/m2 - mean (SD)	26 (5.8)	25.1 (5.0)	26.5 (6.2)
Prior myocardial infarction % (n)	19.4% (56)	18.1% (19)	20.1% (37)
Prior coronary angioplasty % (n)	8% (23)	5.7% (6)	9.2% (17)
Prior Myocardial Revascularization Surgery % (n)	8% (23)	5.7% (6)	9.2% (17)
Smoking % (n)			
Smoker	3.5% (10)	1% (1)	4.9% (9)
Former smoker	25.3% (73)	69.5% (73)	72.3% (133)
Never smoked	71.3% (206)	29.5% (31)	22.8% (42)
Heart rate in bpm - mean (SD)	72 (14.4)	72 (17)	71.6 (12.7)
Systolic blood pressure mmHg - mean (SD)	119.5 (22.9)	113 (20.4)	122 (23.7)
Creatinine mg/dL - mean (SD)	1.14 (0.51)	1.26 (0.7)	1.08 (0.37)
Sodium mEq/L - mean (SD)	139.3 (3.6)	139 (3.27)	139 (3.75)
Potassium mEq/L - mean (SD)	4.6 (0.6)	4.56 (0.57)	4.69 (0.62)
Hemoglobin g/dL- mean (SD)	13.2 (1.6)	13.4 (1.67)	13.1 (1.55)
Concomitant Medications % (n)			
Acetylsalicylic acid	36.7% (106)	38.1% (40)	35.9% (66)
Statin	59.2% (171)	48.6% (51)	65.2% (120)
Anticoagulant	29.4% (85)	31.4% (33)	28.3% (52)
Intracardiac Devices % (n)			
CRT	0.3% (1)	1% (1)	0% (0)
ICD	2.8% (8)	2.9% (3)	2.7% (5)
CRT - D	1% (3)	1.9% (2)	0.5% (1)
Uni or bicameral pacemaker	17.6% (51)	23.8% (25)	14.1% (26)

SD = standard deviation; HF = heart failure; LVEF = left ventricle ejection fraction; NYHA = New York Heart Association; AF = atrial fibrillation; BMI = body mass index; CRT = cardiac resynchronization therapy; ICD = implantable cardioverter-defibrillator; CRT-D = cardiac resynchronization therapy with defibrillation;

*Total sample. For some variables, the total number of observations was smaller.

**Tachycardiomyopathy, peripartum cardiomyopathy, amyloidosis, PRKAG2 mutation, cardiotoxicity.

Chagas and ischemic cardiomyopathies were the main etiologies of HFrEF, corresponding to 27.7% and 26.6% of prevalence, respectively. They were followed by hypertensive etiology with 17%. That prevalences did not change significantly according to LVEF (Table 1). The mean LVEF was 31.5% and most patients (93.8%) were in NYHA class II/III. Most patients (88.6%) with severe heart failure (LVEF < 30%) were also in NYHA class II/III. Table 1 shows the characteristics of the studied sample and according to LVEF, including demographic and clinical aspects, intracardiac device use, medications and comorbidities.

**Figure 1:**
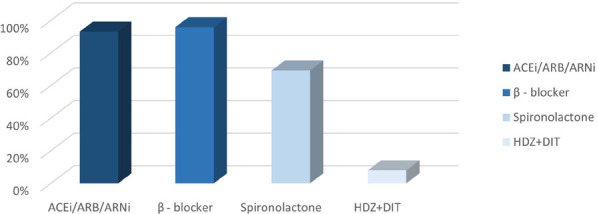
Frequency of prescription of heart failure with reduced ejection fraction drug classes at the outpatient level.

Of the total sample, 93.1% were on ACEi, ARB, or ARNi, with 49.1% on ACEi, 41.2% ARB, and 2.8% ARNi; 95.8% were on beta blockers, 69.2% on spironolactone, and only 8% on the combination hydralazine/isosorbide dinitrate (Figure 1).

ACEi = angiotensin-converting enzyme inhibitor; ARB = angiotensin receptor blocker; ARNI = angiotensin receptor-neprilysin inhibitor; HDZ+DIT = Hydralazine and Isosorbide Dinitrate.

**Figure 2:**
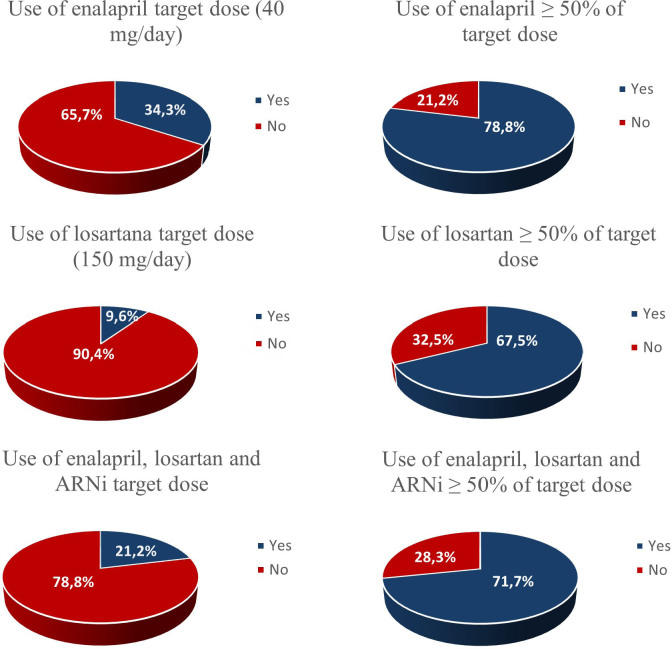
Prescription frequency of enalapril, losartan, and sacubitril/valsartan at target doses and ≥ 50% of the target.

Among users of ACEi, enalapril accounted for 96.5% of prescriptions, while captopril accounted for 2.8% and perindopril only 0.7%. The mean daily dose of enalapril prescribed was 24.6 mg/day, but only 34.3% of enalapril users reached the upper limit of the target dose recommended by the Brazilian guideline, i.e., 40 mg/day.^4^ 78.8% of patients were using enalapril ≥ 50% of the target doses (Figure 2). In bivariate analysis, the variables age, sex, race/ethnicity, HF etiology, NYHA class, DM, CKD and AF did not influence the enalapril prescription dose, so multivariate models were not built.

ARNi = angiotensin receptor-neprilysin inhibitors.

Losartan accounted for 95.8% of prescriptions among ARB users, while 3.4% received valsartan and 0.8% olmesartan. The average dose of losartan prescribed was 88.4 mg/day and only 9.6% reached the upper limit of the target dose recommended by the Brazilian guideline, i.e., 150 mg/day.^4^ 67.5% were using losartan ≥ 50% of the target dose (Figure 2). In bivariate analysis, presence of permanent pacemaker and hypertension were shown to be associated with the prescribed losartan dose, but adjusted ORs did not reach statistical significance in the multivariate model.

Only eight (8) patients were on ARNi in the sample, 75% using the target dose of 400 mg/day and 100% using ≥ 50% of the target dose. Finally, among patients using enalapril, losartan, and ARNi, 21.2% achieved the upper limit of the target dose recommended^4^ and 71.7% achieved ≥ 50% (Figure 2).

**Figure 3:**
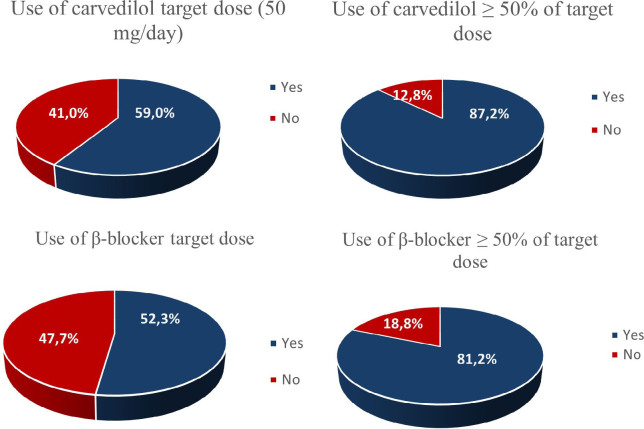
Frequency of prescribing carvedilol and others beta-blockers at target doses and dose ≥ 50% of the target.

Two hundred seventy-seven (277) patients were prescribed beta blockers, with 70.4% represented by carvedilol, with a mean prescriptive dose of 40.4 mg/day. Of the 195 patients taking carvedilol, 53.3% received the target dose of 50 mg/day and 87.2% received ≥ 50% of the target dose (Figure 3). Also, eight (8) patients (4.1%) received the 100 mg/day dose, the target dose recommended for those weighing > 85kg.^13^ Of BB users, 21.7% were prescribed metoprolol succinate, at a mean dose of 101.7 mg/day. 69% were prescribed a dose ≥ 50% of that recommended by guideline and only 17.2% were at the target dose of 200 mg/day.^4^ 19 patients were on bisoprolol (6.9%) and 78.9% had a dose ≥ 50% of the recommended and 36.8% got bisoprolol 10 mg/day (target dose).^4^

The mean dose of bisoprolol prescribed was 6.25 mg/day. Only 0.7% of the sample was on nebivolol, 100% being 5 mg/day (target dose).^4,8^ Finally, the percentage of patients using carvedilol, metoprolol succinate, or bisoprolol at a dose ≥ 50% of the recommended was 81.2% and using target dose, 43.7% (Figure 3).

In the bivariate analysis, younger patients and patients with higher body weight (> 85 kg) were associated with the prescription of a higher dose of carvedilol. Only 3.4% of patients weighing ≤ 85 kg received a dose of carvedilol ≥ 50 mg, compared to 31.8% of those weighing > 85 kg (P < 0.001). Chagasic etiology was associated with higher prevalence of low-dose carvedilol (≤ 25 mg/day) than in non-chagasic patients (54.9% vs. 35.4%, respectively; P = 0.015). On multivariate analysis, the associations between these variables and the prescribed dose of carvedilol lost statistical significance.

**Figure 4:**
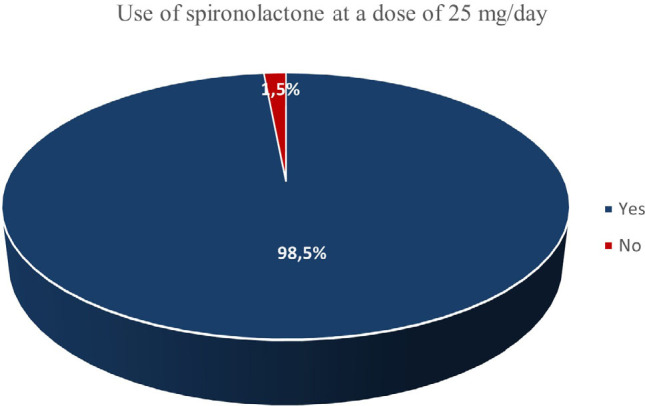
Frequency of prescription of spironolactone at a dose of 25 mg/day.

As much as 69.2% patients had a prescription for MRA in our sample receiving spironolactone with a mean dose of 24.6 mg/day; also 100% were using a dose ≥ 50% of that recommended by guideline,^4^ considering the lower recommendation limit.^4^ In addition, 94% were using the recommended dose of 25 mg/day (Figure 4).

Using spironolactone 50 mg/day, the upper recommendation limit of the Brazilian guideline,^4^ only nine cases (4.5%) were observed. Age, sex, race/ethnicity, HF etiology, NYHA class, DM, CKD, and AF were not associated with the spironolactone prescription dose.

In the study sample, only 8% (23 patients) were prescribed the vasodilator combination hydralazine/isosorbide dinitrate, with a mean dose of 178/52 mg/day, respectively. 52.2% was receiving dose ≥ 50% of that recommended and 8.7% reached the target dose. Overall, only 0.7% of the sample (2/289) was on target dose of the hydralazine/dinitrate combination. Another 16 (5.5%) patients were using the hydralazine/isosorbide mononitrate combination, not recommended by guidelines.^4-7^

**Figure 5:**
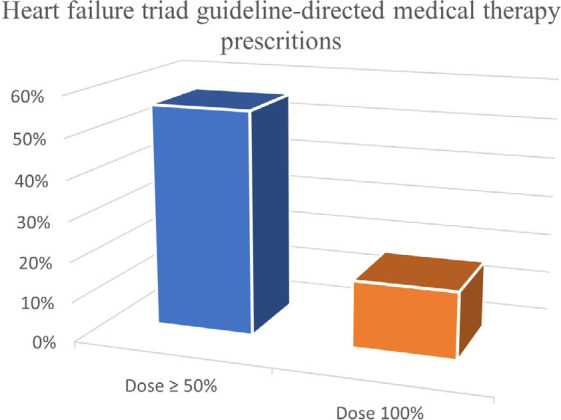
Prevalence of prescriptions of the triad guideline-directed medical therapy for heart failure with reduced ejection fraction ≥ 50% of the recommended and at target doses.

**Figure 6:**
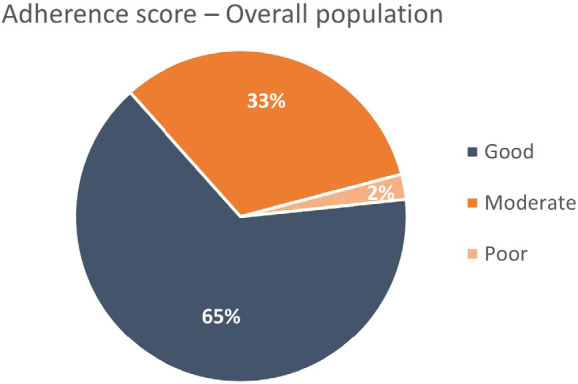
Adherence Score to guideline-directed medical therapy for heart failure with reduced ejection fraction in the overall population.

**Figure 7:**
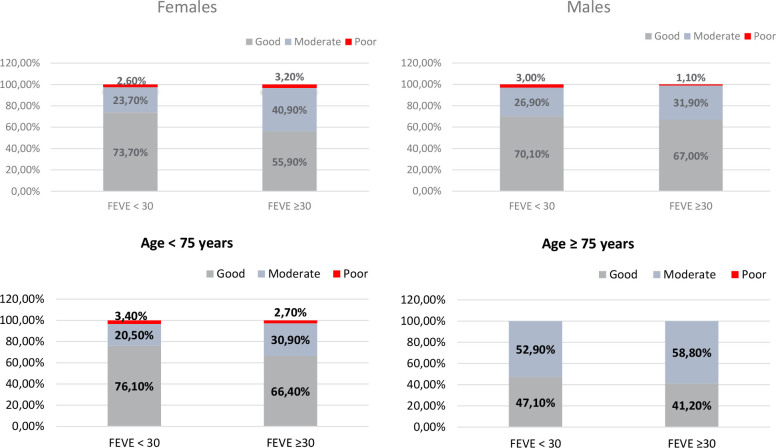
Distribution of different left ventricular ejection fraction categories and guideline adherence scores in heart failure patients by age and sex.

The overall prevalence of the triad GDMT prescriptions were 56% when ≥ 50% of the recommended doses were analyzed and only 17% were on target doses (Figure 5). Based on the adherence score, the percentages of good, moderate, and poor adherence were 65%, 33%, and 2%, respectively, in the overall HF population (Figure 6). No significant difference was found between men and women with good adherence score to GDMT, but, females with LVEF < 30% had better good adherence score than those with LVEF ≥ 30% (Figure 7). Also, younger patients (age < 75 years) had more good adherence score than older ones (≥ 75 years) (Figure 7).

Good = use of three indicated medications from the triad for heart failure with reduced ejection fraction.

Moderate = use of more than half of the indicated medications.

Poor = use of less than half of the indicated medications.

In the population studied, 20.4% of patients were taking digoxin with an average dose of 0.18 mg/day and 66.4% were using the loop diuretic furosemide, with an average dose of 71.9 mg/day. Only 12.8% were using a thiazide diuretic, mainly hydrochlorothiazide (94.6%) with an average dose of 23.9 mg/day; the other 5.4% were using chlorthalidone with an average dose of 18.8 mg/day. Only one patient in the study was on a prescription for ivabradine, at a dose of 10 mg/day. The prescription of sodium-glucose cotransporter 2 (SGLT2i) inhibitors was not evaluated, since, at the time of the survey, this drug class was not recommended by guideline^4^ for use in HFrEF.

## DISCUSSION

We observed, in the present study, based on an outpatient sample, predominantly mixed/afro-descendant, with a high prevalence of Chagas disease as the etiology of HFrEF, and, in its great majority, in class II/III (NYHA), a high prevalence of prescription of GDMT with class I recommendation.

Our data show that 93.1% of patients were using ACEi, ARB, or ARNi, even with a high prevalence of CKD (28%). These results agree with recent studies in HFrEF as in DAPA-HF,^22^ where the use of these classes of drugs reached 94.4%, as well as 89.2% in the EMPEROR-Reduced.^23^ The data found in our institution become relevant when we compare to data of other registries, such as CHAMP-HF^17^ and IMPROVE-HF,^19^ where the baseline frequencies of use were 72.1% and 79.8% respectively.

When we analyze the doses of enalapril, losartan (respectively ACEi and ARB most prescribed in the sample) and ARNi used, we find a nice number of patients using a dose ≥ 50% of the recommended, 71.7%, but this number drops dramatically to 21.2% when we considered 100% of the target dose, number close to that achieved in the CHAMP-HF,^17^ where target doses of enalapril and losartan were not discriminated but the results reported are 16.8% for 100% of the target dose and 40.4% for dose ≥ 50% of the recommended dose.

Enalapril, the most used ACEi in the sample, was prescribed at a mean dose of 24.6 mg/day, a higher dose than the mean found in SOLVD,^10^ which was 16.6 mg/day, and in the recent PARADIGM-HF^24^ cohort, which was 18.9 mg/day. The association of high doses of enalapril with spironolactone has not been adequately evaluated in MRA studies,^16,25^ and concerns about the safety of this association may be a limiting factor for prescribing higher doses of enalapril; however, in the sample studied, we found high doses of enalapril associated with frequent prescription of aldosterone antagonist.

Among ARBs, we found a mean prescribed dose of losartan of 88.4 mg/day, a lower mean dose than that used in HEAAL,^26^ which was 129 mg/day. In the present study, 57.9% of those taking losartan received a dose of 100 mg/day. The main HFrEF guidelines^4-7^ recommend ACEi in preference to ARBs, reserving this class of drugs for cases of intolerance (such as persistent cough) or allergy to ACEi. However, we found a high prevalence of ARB use, very close to that of ACEi, 41.2% (119/289) and 49.1% (142/289), respectively. This finding is not compatible with what has been verified in other HFrEF cohorts, such as in the BREATHE,^2^ QUALIFY^27^ or European Society of Cardiology HFrEF registries,^18^ whose prescription frequencies were 23.5%, 21% and 23.5% for ARBs and 42.2%, 66.2% and 70.7% for ACE inhibitors, respectively.

The high prevalence of ARB prescription evidenced by this study suggests a prescribing habit of cardiologists in the service, a possibility of greater intolerance to ACEi in our population profile, predominantly afro-descendant, or, ultimately, the possibility of easier access to this class of drug. It is worth to consider that we used for all analyses, the upper limit of the target doses of enalapril and losartan recommended by the Brazilian guideline,^4^ which coincides with the recommendations of the North American and European guidelines,^6,7^ i.e., 40 mg/day for enalapril and 150 mg/day for losartan.

The European guideline presents two possible target doses for enalapril, 20 and 40 mg/day, but recommend only a target dose for losartan: 150 mg/day.^7^ We stress, therefore, that the double recommendation of target doses in main HFrEF guidelines^4,6,7^ for drugs known to be effective, such as enalapril, losartan, carvedilol and spironolactone, may make it difficult to assess the adequacy of prescriptions in different health services, and maybe, be an inadequate source of treatment guide, if physicians follow the lower doses recommended. We chose to use 40 mg of enalapril as a target because we considered that 10 mg/day, a dose ≥ 50% of the recommended if the target is 20 mg/day, configures a very low dose to be considered adequate in a population with such high morbidity and mortality. We also opted for a target dose of losartan at 150 mg/day, since this dose in the HEAAL study^26^ was shown to be superior in clinical outcomes than the dose of 50 mg/day.

The low prevalence of ARNi use (2.8%) observed in this study is due, at least in part, to its high cost, considering that the sample consisted exclusively of patients seen at the public health system of Brazil, a middle-income country. Moreover, the sacubitril/valsartan combination had not yet received a favorable opinion from the National Committee for Incorporation of Technologies in the public health system (CONITEC) for incorporation into high-cost medications when the data from this study were collected. The mean dose of sacubitril/valsartan found in the sample was 350 mg/day, close to that found in PARADIGM-HF,^24^ which was 375 mg/day.

The prevalence of beta blocker use in the present study was 95.8%, similar to the DAPA-HF^22^ and EMPEROR-Reduced^23^ studies, respectively 96.1% and 94.7%. In the CHAMP-HF,^17^ IMPROVE-HF^19^ and QUALIFY^27^ registries, the baseline prevalence of beta blocker use was 66.8%, 86.2%, and 86.6%, respectively. In the present sample, 77.8% were on a dose ≥ 50% of the recommended dose, considering carvedilol target dose of 50 mg/day, which is the recommended target dose of carvedilol in the US Carvedilol studies^12,13^ for patients weighing < 85 kg. Only 48.4% of patients were prescribed the target dose.

These numbers should raise the need for strengthening the training of attending physicians to encourage the prescription of appropriate doses of beta blockers, since these findings show room for improvement. However, when compared to the frequency of use of beta blocker in real-life international clinical registries, such as CHAMP-HF^17^ and QUALIFY,^27^ where the use of ≥ 50% of the recommended dose was 54.3% and 52.2%, respectively, it is of notice that our institution shows a pattern of prescription close to the centers participating in such registries. Also, we did find adherence scores of GDMT for HFrEF with a high percentage of good and moderate, respectively 65% and 33%, only 2% poor; while Chen and colleagues, in Sweden, in a larger population studied and not restricted to the university environment, found only 20.1% e 56.9% of good and moderate respectively, while 23% had poor adherence score.^20^ In contrast to Chen et al, we did found more good adherence score to GDMT in more severe heart failure patients (LVEF < 30%), independently of age and sex, but like Chen, more younger patients (< 75 years) had good adherence score to GDMT than older ones (≥ 75 years).^20^

It is also relevant to notice that the northeastern region of Brazil is prevalent for Chagas disease as an etiology of HFrEF, a cause of myocardiopathy commonly associated with lower tolerance to beta blocker, either because of conduction disorders associated with this etiology or because they tend to have lower blood pressure and/or lower prevalence of systemic arterial hypertension as comorbidity.^28^

The prevalence of MRA prescription was high, especially when compared with the HFrEF cohorts from the most recent clinical trials, DAPA-HF^22^ and EMPEROR-Reduced,^23^ where the baseline frequency of use was 71.0% and 71.3%. It should also be noted that 28% of the present sample had CKD, a possible reason for non-prescription. Furthermore, the mean dose of 24.6 mg/day of spironolactone found in the sample was close to that found in the RALES study,^16^ which was 26 mg/day. In the CHAMP-HF,^17^ IMPROVE-HF^19^ and QUALIFY^27^ registries, the baseline prevalence of spironolactone use was 33.1%, 34.4%, and 69.5%, respectively. In CHAMP-HF,^17^ 98.2% were using a dose ≥ 50% of the recommended and 76.6% were using the target dose, values close to but lower than those observed in our sample.

We did not find prevalence data on the use of the hydralazine/dinitrate association in the most recent clinical trial cohorts in HFrEF^22,23^ nor in the registries surveyed^17-19,27^ however, we can state that prescription prevalence of 8%, and with only 52.2% of these with a dose ≥ 50% of the recommended, is a low value, because, this sample was predominantly of mixed people or Afro American (86.5%) and with symptomatic heart failure (99% in CF II-IV, NYHA).

### Limitations

The study limitations are related to its observational, cross-sectional and single-center nature, and also, for being carried out in a cardiology referral service (University Hospital); it is likely that our data do not reflect the reality outside the university environment. As a cross-sectional study, we are subject to selection bias, in which the characteristics or behaviors of the studied sample, may not be representative of the entire target population. In the BREATHE registry,^2^ which included reference institutions in the cardiology area in our country, the rate of use of medications for HF at hospital discharge was much lower than that found in this study, probably noting the differences in the population studied. Also, as a limitations of a cross-sectional study, we are not able to verify causality associations related to the prescription of GDMT, in addition to the fact that we have data collected from medical records, which does not necessarily reflect the medications and doses actually taken by patients. The inferential statistical analyses are of exploratory nature, since sample calculation for a priori hypothesis was not performed. On the other hand, the results found explain real-world clinical practice in a highly complex public health care setting. The knowledge of this reality offers subsidies for the adoption of actions to improve health care for patients with HFrEF, as well as can influence public policies aimed to reduce adverse outcomes related to this serious pathology.

### Future directions

There is need to know how the prescription pattern found impacts mortality in a real world setting and look if the new drug classes recommended by guidelines, namelly ARNi and iSGLT2, are being prescribed and also, if they are able to change, with economic viability and in a real world setting, morbidity and mortality, like they showed in their landmark studies.

## CONCLUSIONS

We found high frequencies of prescription of effective GDMT for HFrEF, but prescription of target doses were low in ACEi, ARB or ARNi and beta-blockers.
